# Treatment Patterns and Clinical Outcomes of Patients with Moderate to Severe Acute Graft-Versus-Host Disease: A Multicenter Chart Review Study

**DOI:** 10.3390/hematolrep16020028

**Published:** 2024-05-06

**Authors:** David Michonneau, Raynier Devillier, Mikko Keränen, Marie Thérèse Rubio, Malin Nicklasson, Hélène Labussière-Wallet, Martin Carre, Anne Huynh, Elisabet Viayna, Montserrat Roset, Jonathan Finzi, Minja Pfeiffer, Daniel Thunström, Núria Lara, Lorenzo Sabatelli, Patrice Chevallier, Maija Itälä-Remes

**Affiliations:** 1 Hôpital Saint-Louis, Université de Paris, 1 Av. Claude Vellefaux, 75010 Paris, France; 2 Institute Paoli-Calmettes, 232 Boulevard de Sainte-Marguerite, 13009 Marseille, France; 3 Helsinki University Hospital, Yliopistonkatu 3, P.O. Box 4, 00014 Helsinki, Finland; 4 Service d’Hématologie, Hôpital Brabois, Centre Hospitalier Régional Universitaire Nancy, Rue du Morvan, 54511 Vandoeuvre-les-Nancy, France; 5 Section of Hematology and Coagulation, Department of Specialist Medicine, Sahlgrenska University Hospital, 413 46 Gothenburg, Sweden; 6 Hôpital Lyon-Sud, 165 Chemin du Grand Revoyet, 69310 Pierre Bénite, France; 7 Centre Hospitalier Universitaire Grenobles Alpes, Av. des Maquis du Grésivaudan, 38700 La Tronche, France; 8 Centre Hospitalier Universitaire Toulouse, l’Institut Universitaire du Cancer de Toulouse-Oncopole, 1 Av. Irène Joliot-Curie, 31059 Toulouse, France; 9 IQVIA Real World Solutions, Provença 392, 3rd Floor, 08025 Barcelona, Spain; 10 Incyte Biosciences France, 35 Ter Avenue André Morizet, 92100 Boulogne-Billancourt, France; 11 Incyte Biosciences International Sàrl, Rue Docteur-Yersin 12, 1110 Morges, Switzerland; 12 Centre Hospitalier Universitaire Nantes, 5 allée de l’Île-Gloriette, 44000 Nantes, France; 13 Turku University Hospital, Kiinamyllynkatu 4-8, 20521 Turku, Finland

**Keywords:** hematopoietic stem cell transplantation, Europe, retrospective, mortality, real-world

## Abstract

Acute graft-versus-host disease (aGVHD) remains a barrier to successful allogeneic hematopoietic stem cell transplantation (HSCT) outcomes. Contemporary comprehensive analyses of real-world clinical outcomes among patients who develop aGVHD post-HSCT are needed to better understand the unmet needs of this patient population. This multicenter, retrospective chart review describes treatment patterns and clinical outcomes among patients (≥18 years old) from Finland, Sweden, and France who developed grades II–IV aGVHD after their first HSCT (January 2016–June 2017). From 13 participating centers, 151 patients were included. The median (Q1, Q3) age at HSCT was 56 (45, 62) years old. One line of aGVHD treatment was received by 47.7%, and the most common first-line treatment was methylprednisolone (alone or in a combination regimen, 74.2%; monotherapy, 25.8%). Among patients treated with methylprednisolone, 79.5% achieved a complete or partial response. The median (Q1, Q3) number of treatment lines was 2.0 (1.0, 3.0). The median (Q1, Q3) time to obtain an aGVHD diagnosis from transplant was 29.5 (21.0, 44.0) days, and 14.5 (7.0, 34.0) days to achieve the best response for 110 evaluable patients. At 6 and 12 months, 53.6% and 49.0%, respectively, achieved a complete response. Chronic GVHD occurred in 37.7% of patients, and aGVHD reoccurred in 26.5%. Following aGVHD diagnosis, mortality rates were 30.0% at 6 months and 37.3% at 12 months. Findings from this study demonstrate a continuing unmet need for new therapies that control aGVHD and improve mortality.

## 1. Introduction

Allogeneic hematopoietic stem cell transplantation (HSCT) is the only potentially curative therapy for hematologic cancers and other diseases of the hematopoietic system [1,2,3]. However, 30% to 60% of allogeneic HSCT recipients develop acute graft-versus-host disease (aGVHD) [4,5,6], which primarily manifests in the skin, liver, and gastrointestinal (GI) tract [7]. aGVHD occurs when alloreactive donor T-cells attack healthy tissue rather than tumor cells [8]. Risk factors for the development of aGVHD include the extent of HLA mismatch (unrelated donor or HLA-mismatched donor), stem cell source (e.g., peripheral blood, bone marrow), sex disparity between donor and recipient, higher-intensity conditioning regimen, and the type of GVHD prophylaxis [8].

Acute GVHD is a potentially life-threatening complication that represents a major cause of non-relapse mortality [9,10]. Furthermore, approximately 35% to 60% of patients do not respond to, or become refractory to, recommended first-line treatment with corticosteroids [4,11,12], with considerably higher mortality rates observed among patients with steroid-resistant disease than in those with steroid-sensitive disease [12].

There are few contemporary comprehensive analyses describing clinical outcomes specifically among patients who develop aGVHD post-HSCT in a real-world setting. In the claims analyses of patients undergoing allogeneic HSCT in the United States (US), higher rates of serious organ toxicities, infections, and inpatient mortality have been reported among patients who develop aGVHD than in HSCT recipients without GVHD [13,14,15]. A retrospective study using Center for International Blood and Marrow Transplant Research (CIBMTR) registry data, from patients with grade II and grade III/IV aGVHD, reported rates of 1-year overall survival (70% and 40%, respectively), transplant-related mortality (12% and 41%), disease-free survival (60% and 34%), and chronic GVHD (cGVHD; 57% and 37%) for the most recent time period analyzed (2006–2012) [16]. Additionally, in a single-center US study of patients who developed grade II–IV aGVHD following HSCT, 18-month survival was 41% among patients who responded to corticosteroid therapy versus 22% among non-responders; 2-year survival was 36% and 17%, respectively [11]. More recently, a retrospective, single-center comparison of HSCT outcomes between 2003 and 2007, and 2013 and 2017, demonstrated that rates of non-relapse mortality (hazard ratio [HR], 0.66), malignancy relapse (HR, 0.76), relapse-related mortality (HR, 0.69), and overall mortality (HR, 0.66), all improved across the decade; however, the overall mortality rate in the 2013–2017 period was 40% [17].

In a prior analysis from the present study, that included only patients from Finland and Sweden with grades II–IV aGVHD, nearly 90% of patients required hospitalization, with a median length of stay of 26 days and over 10 outpatient or emergency department visits per year on average [18]. Patients who received multiple treatment lines had longer hospital stays than those who received only one line of treatment. The aim of this analysis was to describe real-world treatment patterns and clinical outcomes among patients who developed moderate or severe aGVHD following allogeneic HSCT in Finland, Sweden, and France.

## 2. Materials and Methods

### 2.1. Study Design and Patients

This retrospective observational chart review study was originally planned in six European countries (Germany, Italy, Sweden, Finland, United Kingdom, and France). Due to the sponsor’s decision, the study was terminated early and was not initiated in Italy or Germany; an independent study was run in the United Kingdom according to a local protocol and case report form, but the limited data collected were not suitable for inclusion in this analysis. Thus, the study was ultimately conducted in Finland and Sweden (the AGHOS study) and in France (the GRAFITE study). The two studies had a common protocol and study design, with minor differences noted below. The present analysis describes data collected across 13 study centers (two centers in Finland, one in Sweden, and 10 in France).

Eligible patients were ≥18 years old and received allogeneic HSCT from any donor source using bone marrow, peripheral blood stem cells, or umbilical cord blood between 1 January 2016 and 30 June 2017. At least 12 months before data collection, patients were diagnosed with grades II–IV aGVHD per Mount Sinai Acute GVHD International Consortium (MAGIC) criteria [19] or, alternatively, grades II–IV according to the Glucksberg Severity Index or Keystone criteria [20], or grades B–D based on International Bone Marrow Transplant Registry [IBMTR] criteria [21]. In France, only modified Glucksberg criteria were allowed for grading at diagnosis. Comparability across the different grading systems was ensured by assessing aGVHD severity based on the extent of skin, liver, and GI involvement. Grades II–IV aGVHD were defined as skin stage ≥ 3 and/or liver ≥ 1 and/or GI ≥ 1 per MAGIC and modified Glucksberg/Keystone criteria; grades B–D were defined as skin stage ≥ 2 and/or liver ≥ 1 and/or GI ≥ 1 per IBMTR criteria (Supplementary Figure S1) [22]. Patients who were graded based on MAGIC criteria were compared with those graded on alternative scales by using mapping rules derived from grade and organ score definitions for each respective scale (Supplementary Figures S2 and S3) [18].

Patients were required to have medical records containing clinical details of the original disease that led to an HSCT, as well as clinical information on aGVHD presentation and treatment. Patients who developed aGVHD were included retrospectively, beginning with those who received HSCT on 30 June 2017, then working backwards until 1 January 2016. In France, all consecutive patients were identified, and the final cohort for inclusion was randomly selected. In Finland and Sweden, patients were included up until 1 January 2016, or until the target sample size was reached (whichever occurred first). A sample size of 135 patients was predefined to describe continuous and categorical variables with a predefined precision level of 0.06; 13% was the maximum percentage of patients with non-available data due to the retrospective data. The coefficient of variation (standard deviation [SD]/mean) considered was 0.5, with 95% CI for continuous variables corresponding to 0.128. For categorical variables, an occurrence rate of 50% was considered with a precision level of 6%.

### 2.2. Data Collection

Patient medical records were reviewed from the index date (date of allogeneic HSCT) until the day of data collection or until death or lost to follow-up (whichever occurred first). Detailed patient demographics, transplant characteristics, HSCT characteristics and clinical presentation, treatments, and outcomes of aGVHD were abstracted from medical records retrospectively and entered into electronic case report forms. Disease diagnoses (e.g., comorbidities) and medical procedures were coded per the Medical Dictionary for Regulatory Activities.

### 2.3. Ethical Considerations

The study was conducted in accordance with the study protocol, the Declaration of Helsinki, and all relevant regulatory requirements. Independent ethics committees/institutional review boards approved the study protocol prior to patient enrollment. The GRAFITE study was conducted in compliance with MR-004 in France. Personal data were processed in accordance with the EU General Data Protection Regulation.

### 2.4. Statistical Analyses

All statistical analyses were conducted using SAS^®^ version 9.2 or higher (SAS Institute, Cary, NC, USA). Data were summarized using descriptive statistics and reported for the overall population and stratified by country. Continuous variables were reported as mean (SD) or median (Q1, Q3); categorical values were summarized as frequency and percentage of the total study population, and by subgroups where appropriate. A prespecified subgroup analysis was conducted, based on the prophylactic regimen received, to evaluate clinical outcomes (e.g., time from HSCT to aGVHD diagnosis, best overall response, cGVHD development, recurrence of GVHD).

## 3. Results

### 3.1. Patients

Data from 13 participating centers (Finland, *n* = 2; Sweden, *n* = 1; France, *n* = 10) were included in this analysis; the majority of sites (*n* = 11; 84.6%) were academic/university centers; seven (53.8%) were public medical centers and one (7.7%) was a specialized cancer center (Table 1). Overall, sites conducted a median (Q1, Q3) of 91 (70, 115) adult transplants per center during the study period. A total of 427 patients (Finland, *n* = 46; Sweden, *n* = 10; France, *n* = 371) developed grades II–IV aGVHD post-HSCT between 1 January 2016 and 30 June 2017. This analysis included a subset of 151 patients (Finland, *n* = 45; Sweden, *n* = 10; France, *n* = 96) who met all eligibility criteria. Overall, 95 (62.9%) patients were male; the median (Q1, Q3) age was 55.5 (45.0, 62.0) years both at transplant and at grade II–IV aGVHD diagnosis (Table 2). Acute myeloid leukemia was the most common indication for HSCT (*n* = 52 [34.4%]), followed by acute lymphocytic leukemia (*n* = 23 [15.2%]). At the time of transplant, 93 (61.6%) patients were in complete remission, and 21 (13.9%) had an active relapse or progressive disease. Overall, 44 (29.1%) patients had a disease risk index (DRI) of intermediate; however, DRI was unreported in most of the French population (*n* = 70 [72.9%]) and in over half of the total population (*n* = 76 [50.3%]). Most patients received a graft from an unrelated donor (*n* = 93 [61.6%]), and peripheral blood was the most common stem cell source (*n* = 130 [86.1%]). Overall, 107 (70.9%) patients received a fludarabine-based conditioning regimen, and the most common aGVHD prophylaxis used was cyclosporine (*n* = 128 [84.8%]), followed by mycophenolate (*n* = 83 [55.0%]). Although centers had reported the use of other scales at the site level, MAGIC (*n* = 29 [19.2%]) and modified Glucksberg (*n* = 122 [80.8%]) were the only two scales used for grading aGVHD severity in the study patients. Based on mapping between scales, 85 (56.3%) patients had grade II aGVHD and 46 (30.5%) patients had grade III/IV aGVHD at diagnosis (Supplementary Table S1); 12 (7.9%) patients had grade I aGVHD at diagnosis, and the grade was unknown in eight (5.3%) patients. Regarding aGVHD organ involvement, skin was the most commonly involved organ (*n* = 108 [71.5%]), followed by the upper GI tract (*n* = 53 [35.1%]).

### 3.2. Treatment Patterns

**Figure 1:**
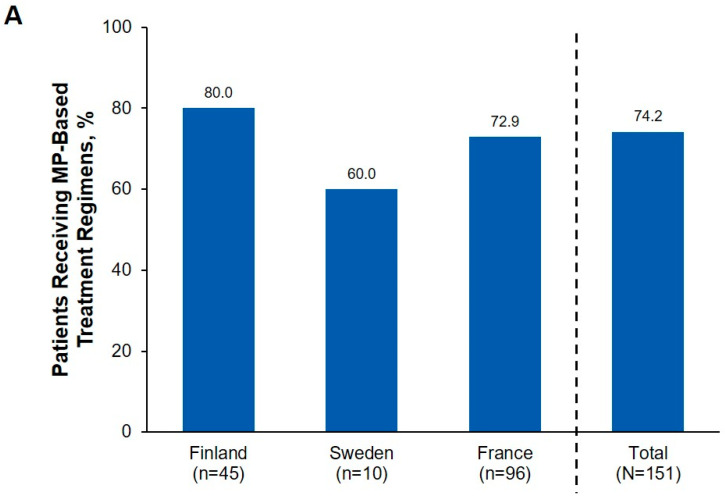

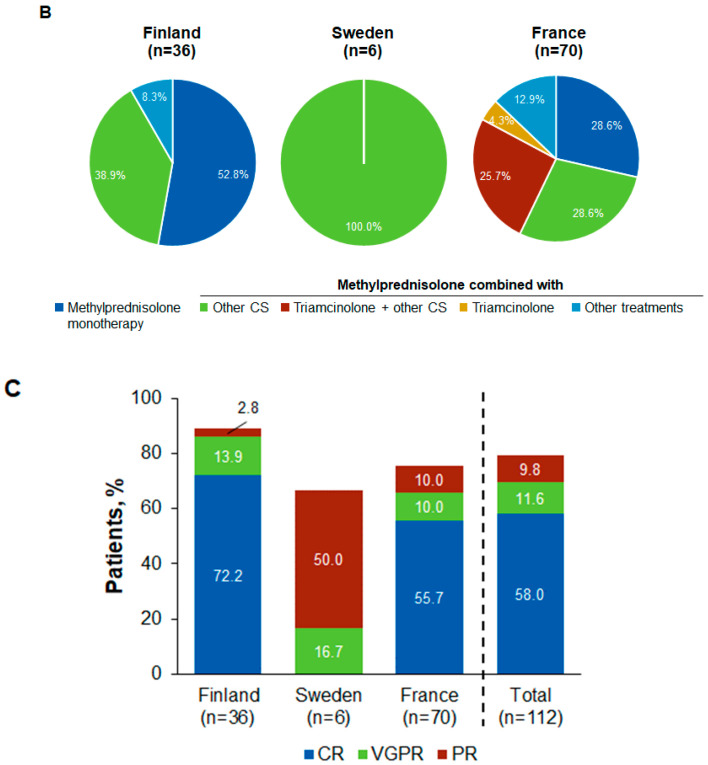
First-line Treatment of aGVHD: (**A**) Percentage of Patients Receiving Methylprednisolone-based Treatment Regimens (Monotherapy or Combination Therapy), (**B**) Methylprednisolone-containing Treatment Regimens,^1^ and (**C**) Response to First-Line Treatment with Methylprednisolone.^2^ aGVHD, acute graft-versus-host disease; CR, complete response; CS, corticosteroids; MP, methylprednisolone; PR, partial response; VGPR, very good partial response. ^1^ Percentages of patients who received each type of regimen^2^ as monotherapy or combined with any other treatments.

Many patients received one line of aGVHD treatment (72 [47.7%]), with 36 (23.8%) and 41 (27.2%) receiving two and three or more lines, respectively (Table 3). The most common first-line treatment was methylprednisolone, which was administered alone or combined with other therapies in 112 (74.2%) patients (Figure 1); 39 (25.8%) patients received methylprednisolone monotherapy overall.

Among the 112 patients who received methylprednisolone alone or in any combination, 65 (58.0%) achieved a complete response and 89 (79.5%) achieved a complete or partial response (Figure 1). Among patients treated with triamcinolone alone or in any combination (*n* = 40; all in France), 29 (72.5%) achieved a complete response and 36 (90.0%) achieved a complete or partial response. The median (Q1, Q3) duration of treatment was 9.0 (5.0, 40.0) days for first-line treatment, and 21.0 (12.0, 38.0) and 39.5 (19.0, 79.0) days for second- and third-line treatment, respectively (Table 3). The median (Q1, Q3) total duration of aGVHD therapy (i.e., including all treatment lines) was 131.0 (71.0, 208.0) days.

### 3.3. Clinical Outcomes

The median (Q1, Q3) time to obtain an aGVHD diagnosis from transplant was 29.5 (21.0, 44.0) days overall. Among 110 evaluable patients, the median (Q1, Q3) time to achieve the best response to aGVHD therapy was 14.5 (7.0, 34.0) days. A complete response was achieved by 81 (53.6%) patients at 6 months and 74 (49.0%) patients at 12 months. Overall, 57 (37.7%) patients developed cGVHD after aGVHD. Additionally, 40 (26.5%) patients overall experienced aGVHD recurrence.

### 3.4. Subgroup Analysis

A subgroup analysis assessed clinical outcomes stratified by the prophylaxis regimen received (Table 4). Among patients who received the most common prophylactic regimen of cyclosporine and mycophenolate (*n* = 40), the median (Q1, Q3) time from transplant to aGVHD diagnosis was 28.0 (17.5, 35.0) days. Patients treated with a combination of in vivo T-cell depletion, cyclosporine, and mycophenolate (*n* = 16) as prophylaxis had the shortest median [Q1, Q3] time for an aGVHD diagnosis (23.0 [15.0, 36.0] days), whereas those treated with in vivo T-cell depletion, cyclosporine, and methotrexate (*n* = 15) had the longest median [Q1, Q3] time for an aGVHD diagnosis (57.0 [33.0, 76.0] days). Among patients who received prophylaxis with cyclosporine and mycophenolate, a complete response was achieved by 17 (42.5%) and 15 (37.5%) of 40 patients at 6 months and 12 months, respectively. The highest rate of complete response was observed among the 15 patients who received prophylactic in vivo T-cell depletion, cyclosporine, and methotrexate (6 months, *n* = 11 [73.3%]; 12 months, *n* = 12 [80.0%]). Among 40 patients who received prophylaxis with cyclosporine and mycophenolate, 11 (27.5%) developed cGVHD following aGVHD. Rates of cGVHD development were highest among the 18 patients who received methotrexate and mycophenolate combination prophylaxis (*n* = 9 [50.0%]) and lowest among the 13 patients who received mycophenolate alone (*n* = 3 [23.1%]). Additionally, of the 40 patients who received prophylactic cyclosporine plus mycophenolate, 15 (37.5%) had aGVHD recurrence, which was the highest percentage across the prophylactic regimen subgroups. Lastly, of the 15 patients who received in vivo T-cell depletion, cyclosporine, and methotrexate, aGVHD recurrence was observed in two patients (13.3%), which was the lowest rate of aGVHD recurrence.

### 3.5. All-Cause Mortality

**Figure 2:**
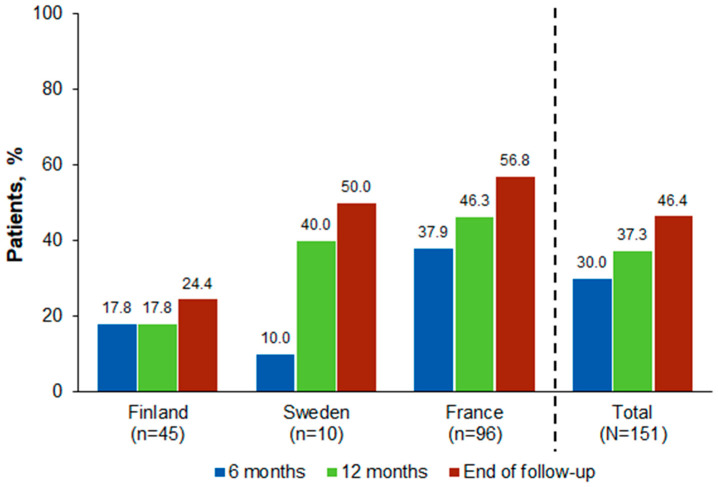
All-Cause Mortality at 6 and 12 Months and End of Follow-Up Since aGVHD Development. aGVHD, acute graft-versus-host disease.

Overall, 45 patients (30.0%) died from any cause at 6 months after aGVHD diagnosis, and 56 (37.3%) died at 12 months (Figure 2).

## 4. Discussion

This real-world study provides a comprehensive overview of the clinical characteristics, treatment patterns, and clinical outcomes of patients who developed aGVHD following allogeneic HSCT in real-world settings in three European countries. Most patients received first-line treatment for aGVHD with methylprednisolone-containing regimens, although variations were seen in the treatment approaches across countries. The overall response rate to these agents in the first-line setting was nearly 80%. However, the patients in this study population were ultimately heavily treated for aGVHD, with over one-quarter receiving three or more lines of treatment. More than one-third of patients developed cGVHD following aGVHD, and nearly one-third of patients in the study died from any cause within 6 months of their aGVHD diagnosis. There may be many reasons for the apparently lower mortality rate among patients from Finland vs. Sweden or France. Since the data reflect all-cause mortality, one possible explanation is that there could have been more patients with mild aGVHD in the Finnish cohort and more patients with severe aGVHD in the French cohort, with excess deaths owing to the procedure rather than disease relapse. Further, it is the authors’ understanding that treatment-related mortality is generally relatively low in Finnish centers, which may reflect pretreatment practices (e.g., the potentially greater utilization of treosulfan there than in other centers).

Findings from this study support the limited previous literature showing poor outcomes among patients who develop aGVHD post-HSCT, including several retrospective claims analyses conducted in the US [13,14,15,23]. These previous studies demonstrated that patients who developed aGVHD following HSCT experienced an increased incidence of serious organ system conditions and infections, higher healthcare resource utilization and costs, and greater mortality than those who did not develop aGVHD. While the most common first-line treatment with methylprednisolone in the current study was similar to that utilized in a 2014–2016 US-based chart review (corticosteroids, 97.9%) [23], aGVHD prophylaxis received in the current study (cyclosporine 84.8%; mycophenolate 55.0%) differed from that administered in US-based studies, which showed that the use of tacrolimus as aGVHD prophylaxis has grown since 1999–2002, virtually replacing cyclosporine during the 2006–2012 period (27% vs. 72% and 80% vs. 17%, respectively) [16]. Additionally, in a US-based chart review, the majority of patients who received allogeneic HSCT between 2014 and 2016 received tacrolimus-based GVHD prophylaxis (56.4%) rather than cyclosporine or mycophenolate (26.1% and 44.0%, respectively) [23].

Although findings were not compared against a non-GVHD cohort in the current study, substantial all-cause mortality was observed among patients who developed aGVHD after HSCT. In the current analysis, at the end of the first year after aGVHD diagnosis, 63% of patients were alive, which fell within the range reported in a prior CIBMTR study (40–70% 1-year survival, depending on aGVHD grade) [16]. Mortality rates in the current study were also similar to 2006–2012 1-year mortality rates in a US-based study (Grade II aGVHD: 30%; Grade III–IV aGVHD: 60%) [16], and in the 2014–2016 US-based chart review (Grade II aGVHD: 31.8%; Grade III–IV aGVHD: 41.7%) [23].

Our group has previously shown that moderate to severe aGVHD is associated with high rates of hospitalizations and outpatient visits among patients receiving allogeneic HSCT at transplant centers in Finland and Sweden [18]. Taken together, findings from this study and previous reports demonstrate the substantial burden of aGVHD among allogeneic HSCT recipients and highlight the need for more effective treatment strategies that can improve clinical outcomes.

Several limitations should be noted. First, this was a retrospective chart review study with potential for inaccurate or missing data entries. Additionally, the study was characterized by a small sample size, particularly in the Nordic populations. Finally, study data are heterogeneous due to several reasons, such as differences between centers in aGVHD diagnosis, treatment of aGVHD, and numbers of recruited patients. Variability existed in patient selection across countries, which may have influenced clinical outcomes. The differences in staging and grading systems among sites (i.e., MAGIC vs. modified Glucksberg) may have affected the results, although an alignment process was performed to ensure compatibility across grading systems (Supplementary Figures S1–S3). The alignment process grouped and mapped modified Glucksberg cases into MAGIC (instead of doing the opposite, i.e., mapping or naively comparing patients graded in MAGIC with patients graded in Glucksberg) [18].

## 5. Conclusions

In conclusion, moderate to severe aGVHD post-HSCT is associated with poor clinical outcomes, including the need for multiple lines of treatment, the development of cGVHD, and high mortality rates. Additional prospective studies covering a greater geographic domain are needed to better understand the widespread burden of aGVHD among patients receiving allogeneic HSCT for hematologic malignancies and disorders.

## Figures and Tables

**Table 1:** Characteristics of Participating Transplant Centers.

Characteristic	Finland (*n* = 2)	Sweden (*n* = 1)	France (*n* = 10)	Total (*n* = 13)
Type of transplant center, *n* (%) ^1^				
Academic/university	2 (100.0)	1 (100.0)	8 (80.0)	11 (84.6)
Public	1 (50.0)	0	6 (60.0)	7 (53.8)
Specialized cancer center	0	0	1 (10.0)	1 (7.7)
Physicians dedicated to HSCT (≥90% of the time), median (Q1, Q3), *n*	3.5 (3.0, 4.0)	3.0 (3.0, 3.0)	4.5 (3.0, 7.0)	4.0 (3.0, 5.0)
Adult HSCTs conducted (1 January 2016–30 June 2017), median (Q1, Q3), *n*	95.5 (76.0, 115.0)	53.0 (53.0, 53.0)	94.0 (70.0, 120.0)	91.0 (70.0, 115.0)
Criteria used to grade aGVHD, *n* (%) ^1^				
MAGIC	1 (50.0)	0	2 (20.0)	3 (23.1)
Original Glucksberg	0	0	6 (60.0)	6 (46.2)
Modified Glucksberg or Keystone	1 (50.0)	1 (100.0)	5 (50.0)	7 (53.8)
IBMTR	0	0	1 (10.0)	1 (7.7)
Other	0	0	1 (10.0)	1 (7.7)
Patients with severe (grades III/IV) aGVHD after allogeneic HSCT, median (Q1, Q3), %	34.3 (18.5, 50.0)	25.0 (25.0, 25.0)	30.0 (30.0, 50.0)	30.0 (30.0, 50.0)

aGVHD, acute graft-versus-host disease; HSCT, hematopoietic stem cell transplantation; IBMTR, International Bone Marrow Transplant Registry; MAGIC, Mount Sinai Acute GVHD International Consortium.^1^ Categories are not mutually exclusive.

**Table 2:** Patient Demographics and Clinical Characteristics.

Characteristic	Finland (*n* = 45)	Sweden (*n* = 10)	France (*n* = 96)	Total (*n* = 151)
Sex, *n* (%) ^1^				
Male	25 (55.6)	5 (50.0)	65 (67.7)	95 (62.9)
Female	20 (44.4)	5 (50.0)	30 (31.3)	55 (36.4)
Age at transplant, median (Q1, Q3), y ^1^	54.0 (45.0, 59.0)	44.5 (36.0, 61.0)	56.0 (46.0, 65.0)	55.5 (45.0, 62.0)
Age at grade II–IV aGVHD diagnosis, median (Q1, Q3), y	54.0 (45.0, 59.0)	44.5 (36.0, 61.0)	56.0 (46.0, 65.0)	55.5 (45.0, 62.0)
BMI, median (Q1, Q3), kg/m ^2,3^	27.8 (23.4, 29.6)	25.4 (24.5, 26.5)	25.0 (22.1, 27.2)	25.4 (22.7, 27.9)
Primary disease diagnosis, *n* (%)				
AML	16 (35.6)	3 (30.0)	33 (34.4)	52 (34.4)
ALL	3 (6.7)	1 (10.0)	19 (19.8)	23 (15.2)
MDS	4 (8.9)	0	11 (11.5)	15 (9.9)
MM	8 (17.8)	0	1 (1.0)	9 (6.0)
Hodgkin lymphoma	3 (6.7)	1 (10.0)	4 (4.2)	8 (5.3)
B-cell lymphoma	3 (6.7)	2 (20.0)	0	5 (3.3)
Non–CML MPN	4 (8.9)	1 (10.0)	0	5 (3.3)
CML	1 (2.2)	0	2 (2.1)	3 (2.0)
T-cell lymphoma	0	1 (10.0)	0	1 (0.7)
Other malignancy ^2^	3 (6.7)	1 (10.0)	25 (26.0)	29 (19.2)
Unknown	0	0	1 (1.0)	1 (0.7)
Stage at transplant, *n* (%)				
Complete remission	29 (64.4)	5 (50.0)	59 (61.5)	93 (61.6)
Partial remission	9 (20.0)	3 (30.0)	5 (5.2)	17 (11.3)
Active relapse or progressive disease	5 (11.1)	1 (10.0)	15 (15.6)	21 (13.9)
Patient untreated	2 (4.4)	1 (10.0)	0	3 (2.0)
Unknown/other	0	0	17 (17.7)	17 (11.3)
Disease risk index, *n* (%)				
Low	8 (17.8)	2 (20.0)	4 (4.2)	14 (9.3)
Intermediate	21 (46.7)	6 (60.0)	17 (17.7)	44 (29.1)
High	9 (20.0)	2 (20.0)	4 (4.2)	15 (9.9)
Very high	1 (2.2)	0	1 (1.0)	2 (1.3)
Unknown	6 (13.3)	0	70 (72.9)	76 (50.3)
Conditioning regimen, *n* (%)				
Fludarabine-based	32 (71.1)	9 (90.0)	66 (68.8)	107 (70.9)
Busulfan-based	7 (15.6)	4 (40.0)	54 (56.3)	65 (43.0)
TBI-based	14 (31.1)	1 (10.0)	23 (24.0)	38 (25.2)
Other	3 (6.7)	7 (70.0)	73 (76.0)	83 (55.0)
Donor type, *n* (%)				
Related donor	8 (17.8)	5 (50.0)	44 (45.8)	57 (37.7)
Fully HLA-matched twin	5 (62.5)	0	0	5 (8.8)
Haploidentical donor	0	1 (20.0)	17 (38.6)	18 (31.6)
HLA-matched related donor	3 (37.5)	4 (80.0)	27 (61.4)	34 (59.6)
Unrelated donor	37 (82.2)	5 (50.0)	51 (53.1)	93 (61.6)
HLA matched	35 (94.6)	5 (100.0)	43 (84.3)	83 (89.2)
HLA mismatched	2 (5.4)	0	7 (13.7)	9 (9.7)
Unknown	0	0	1 (1.0)	1 (0.7)

aGVHD, acute graft-versus-host disease; ALL, acute lymphocytic leukemia; AML, acute myeloid leukemia; BMI, body mass index; CML, chronic myeloid leukemia; HLA, human leukocyte antigen; MDS, myelodysplastic syndrome; MM, multiple myeloma; MPN, myeloproliferative neoplasm; TBI, total body irradiation.^1^ Data were missing for one patient from France. ^2^ Data were missing for five patients from Finland and one from France. ^3^ Includes blastic plasmacytoid dendritic cell neoplasia (Finland, *n* = 1), CML (Finland, *n* = 1), MDS (Finland, *n* = 1), and chronic myelomonocytic leukemia (Sweden, *n* = 1); specifications were not reported in France.

**Table 3:** aGVHD Treatment Patterns.

Characteristic	Finland (*n* = 45)		Sweden (*n* = 10)		France (*n* = 96)		Total (*n* = 151)	
Number of treatment lines								
Median (Q1, Q3)	2.0 (1.0–3.0)		2.0 (1.0–3.0)		1.0 (1.0–3.0) ^1^		2.0 (1.0–3.0)	
Distribution, *n* (%)								
1	20 (44.4)		4 (40.0)		48 (50.0)		72 (47.7)	
2	13 (28.9)		3 (30.0)		20 (20.8)		36 (23.8)	
3	7 (15.6)		1 (10.0)		15 (15.6)		23 (15.2)	
4	3 (6.7)		1 (10.0)		4 (4.2)		8 (5.3)	
5	1 (2.2)		1 (10.0)		4 (4.2)		6 (4.0)	
≥6	1 (2.2)		0		3 (3.1)		4 (2.6)	
Unknown	0		0		2 (2.1)		2 (1.3)	
Duration of treatment, median (Q1, Q3), d	*n*		*n*		*n*		*n*	
First line	25	6.0 (5.0, 18.0)	6	25.5 (8.0, 87.0)	20	17.5 (4.5, 60.5)	51	9.0 (5.0, 40.0)
Second line	12	19.5 (12.5, 33.5)	3	20.0 (8.0, 102.0)	7	27.0 (4.0, 72.0)	22	21.0 (12.0, 38.0)
Third line	5	31.0 (19.0, 79.0)	2	78.0 (48.0, 108.0)	3	30.0 (11.0, 60.0)	10	39.5 (19.0, 79.0)
Fourth line	2	59.0 (8.0, 110.0)	1	306.0 (306.0, 306.0)	3	36.0 (9.0, 40.0)	6	38.0 (9.0, 110.0)
Fifth line	1	211.0 (211.0, 211.0)	0	N/A	1	88.0 (88.0, 88.0)	2	149.5 (88.0–211.0)
Total duration of therapy, median (Q1, Q3), d	40	169.5 (86.0, 260.0)	6	196.0 (153.0, 227.0)	43	86.0 (45.0, 169.0)	89	131.0 (71.0, 208.0)
Treatment response of CR, VGPR, or PR, *n* (%) ^2^	*n*		*n*		*n*		*n*	
Methylprednisolone-based therapy	36	32 (88.9)	6	4 (66.7)	70	53 (75.7)	112	89 (79.5)
Triamcinolone-based therapy	0	–	0	–	40	36 (90.0)	40	36 (90.0)
Other corticosteroids	21	19 (90.5)	10	8 (80.0)	64	56 (87.5)	95	83 (87.4)

aGVHD, acute graft-versus-host disease; CR, complete response; N/A, not applicable; PR, partial response; VGPR, very good partial response.^1^ Two patients from France had missing treatment line data; median data are based on *n* = 94. ^2^ Percentages based on number of patients who received specific treatment.

**Table 4:** Subgroup Analysis of Clinical Outcomes by Prophylactic Treatment Regimen. ^1^

Outcome	Cyclosporine + Mycophenolate (*n* = 40)	In Vivo T-Cell Depletion + Cyclosporine + Methotrexate (*n* = 15)	In Vivo T-Cell Depletion + Cyclosporine + Mycophenolate (*n* = 16)	In Vivo T-Cell Depletion + Methotrexate + Mycophenolate (*n* = 25)	Methotrexate + Mycophenolate (*n* = 18)	Mycophenolate (*n* = 13)
Time from HSCT to aGVHD diagnosis						
Median (Q1, Q3), d	28.0 (17.5, 35.0)	57.0 (33.0, 76.0)	23.0 (15.0, 36.0)	34.0 (24.0, 44.0)	25.5 (20.0, 40.0)	30.0 (27.0, 49.0)
Best overall response, *n* (%)						
6 months						
CR	17 (42.5)	11 (73.3)	7 (43.8)	16 (64.0)	8 (44.4)	7 (53.8)
VGPR	0	2 (13.3)	1 (6.3)	1 (4.0)	1 (5.6)	0
PR	3 (7.5)	1 (6.7)	1 (6.3)	1 (4.0)	1 (5.6)	1 (7.7)
Mixed disease	0	0	0	1 (4.0)	1 (5.6)	0
PD	0	0	1 (6.3)	0	0	0
No response	2 (5.0)	0	0	0	0	0
NA/missing	18 (45.0)	1 (6.7)	6 (37.5)	6 (24.0)	7 (38.9)	5 (38.5)
12 months						
CR	15 (37.5)	12 (80.0)	5 (31.3)	15 (60.0)	8 (44.4)	5 (38.5)
VGPR	1 (2.5)	1 (6.7)	1 (6.3)	0	0	0
PR	0	1 (6.7)	1 (6.3)	0	0	0
Mixed disease	0	0	1 (6.3)	1 (4.0)	1 (5.6)	0
PD	1 (2.5)	0	0	0	0	0
No response	1 (2.5)	0	0	0	0	0
NA/missing	22 (55.0)	1 (6.7)	8 (50.0)	9 (36.0)	9 (50.0)	8 (61.5)
Developed cGVHD, *n* (%)						
Yes	11 (27.5)	7 (46.7)	6 (37.5)	10 (40.0)	9 (50.0)	3 (23.1)
NA/missing	4 (10.0)	0	0	0	1 (5.6)	3 (23.1)
aGVHD recurrence, *n* (%)						
Yes	15 (37.5)	2 (13.3)	4 (25.0)	5 (20.0)	6 (33.3)	3 (23.1)
NA/missing	0	1 (6.7)	0	0	0	0

aGVHD, acute graft-versus-host disease; cGVHD, chronic graft-versus-host disease; CR, complete response; HSCT, hematopoietic stem cell transplantation; NA, not available; PD, progressive disease; PR, partial response; VGPR, very good partial response. ^1^ Twenty-three patients received “other” prophylactic regimens and are not shown in the table.

## Data Availability

Access to individual patient-level data is not available for this study. Information on Incyte’s clinical trial data sharing policy and instructions for submitting clinical trial data requests are available at: https://www.incyte.com/Portals/0/Assets/Compliance%20and%20Transparency/clinical-trial-data-sharing.pdf?ver=2020-05-21-132838-960 (accessed on 18 October 2023).

## Supplementary Materials

The following supporting information can be downloaded at: https://www.mdpi.com/article/10.3390/hematolrep16020028/s1, Figure S1: Comparison of (A) aGVHD Severity Grading and (B) Organ Staging Across Grading Scales; Figure S2: Mapping Rules for Comparing MAGIC Grading to Other Scales; Figure S3: Overview of aGVHD Grading Systems; Table S1: aGVHD Severity at Diagnosis Based on MAGIC and mGlucksberg Criteria. References [18,19,20,21,22] are cited in the Supplementary Materials.
