# Supplementary material for: Treatment Patterns and Clinical Outcomes of Patients with Moderate to Severe Acute Graft-Versus-Host Disease: A Multicenter Chart Review Study
Source: Hematol Rep. 2024 May 6;16(2):283–94. doi: 10.3390/hematolrep16020028 (PMC11130792; doi:10.3390/hematolrep16020028)
Supplement: Supplementary file 1 [file hematolrep-16-00028-s001.zip › hematolrep-2821435-supplementary.pdf]

Supplementary Appendix

Treatment Patterns and Clinical Outcomes of Patients with Moderate to Severe Acute Graft-Versus-Host Disease: A Multicenter Chart Review Study

David Michonneau [1,*,†], Raynier Devillier [2,†], Mikko Keränen [3,†], Marie Thérèse Rubio [4], Malin Nicklasson [5], Hélène Labussière-Wallet [6], Martin Carre [7], Anne Huynh [8], Elisabet Viayna [9], Montserrat Roset [9], Jonathan Finzi [10], Minja Pfeiffer [11], Daniel Thunström [11], Núria Lara [9], Lorenzo Sabatelli [11], Patrice Chevallier [12,‡] and Maija Itälä-Remes [13,‡]

[1] Hôpital Saint-Louis, 1 Av. Claude Vellefaux, Université de Paris, 75010 Paris, France
[2] Institute Paoli-Calmettes, 232 Boulevard de Sainte-Marguerite, 13009 Marseille, France
[3] Helsinki University Hospital, Yliopistonkatu 3, P.O. Box 4, 00014 Helsinki, Finland
[4] Service d'Hématologie, Hôpital Brabois, Centre Hospitalier Régional Universitaire Nancy, Rue du Morvan, 54511 Vandoeuvre-les-Nancy, France
[5] Section of Hematology and Coagulation, Department of Specialist Medicine, Sahlgrenska University Hospital, 413 46 Gothenburg, Sweden
[6] Hôpital Lyon-Sud, 165 Chemin du Grand Revoyet, 69310 Pierre Bénite, France
[7] Centre Hospitalier Universitaire Grenobles Alpes, Av. des Maquis du Grésivaudan, 38700 La Tronche, France
[8] Centre Hospitalier Universitaire Toulouse, l'Institut Universitaire du Cancer de Toulouse-Oncopole, 1 Av. Irène Joliot-Curie, 31059 Toulouse, France
[9] IQVIA Real World Solutions, Provença 392, 3rd Floor, 08025 Barcelona, Spain
[10] Incyte Biosciences France, 35 Ter Avenue André Morizet, 92100 Boulogne-Billancourt, France
[11] Incyte Biosciences International Sàrl, Rue Docteur-Yersin 12, 1110 Morges, Switzerland
[12] Centre Hospitalier Universitaire Nantes, 5 allée de l'Île-Gloriette, 44000 Nantes, France
[13] Turku University Hospital, Kiinamyllynkatu 4-8, 20521 Turku, Finland
* Correspondence: david.michonneau@aphp.fr
† These authors contributed equally to this work and are co-lead authors.
‡ These authors contributed equally to this work and are co-senior authors.

Contents

SUPPLEMENTARY FIGURES....2

Supplementary Figure S1. Comparison of (A) aGVHD Severity Grading and (B) Organ Staging Across Grading Scales....2

Supplementary Figure S2. Mapping Rules for Comparing MAGIC Grading to Other Scales....5

Supplementary Figure S3. Overview of aGVHD Grading Systems....6

SUPPLEMENTARY TABLES....7

Supplementary Table S1. aGVHD Severity at Diagnosis Based on MAGIC and mGlucksberg Criteria....7

SUPPLEMENTARY FIGURES

Supplementary Figure S1 Comparison of (A) aGVHD Severity Grading and (B) Organ Staging Across Grading Scales

A

MAGIC Criteria[1,2]

Grade	Organ Involvement
0	Skin=0; liver=0; GI=0
I	Skin=1–2; liver=0; GI=0
II	Skin=3 and/or liver=1 and/or GI=1
III	Skin=0–3; liver=2–3 and/or GI=2–3
IV	Skin=4 and/or liver=4 and/or GI=4

Modified Glucksberg/Keystone Criteria[1,3]

Grade	Organ Involvement
0	Skin=0; liver=0; GI=0
I	Skin=1–2; liver=0; GI=0
II	Skin=3 and/or liver=1 and/or GI=1
III	Skin=0–3; liver=2–3 and/or GI=2–4
IV	Skin=4 and/or liver=4; GI=0–4

IBMTR Criteria[4]

Grade	Organ Involvement
0	Skin=0; liver=0; GI=0
A	Skin=1; liver=0; GI=0
B	Skin=2 and/or liver=1–2 and/or GI=1–2
C	Skin=3 and/or liver=3 and/or GI=3
D	Skin=4 and/or liver=4 and/or GI=4

B

MAGIC Criteria[1,2]				
Stage	Skin	Liver	Upper GI	Lower GI
0	No rash	Bilirubin <2 mg/dL	No or intermittent nausea, vomiting, or anorexia	Diarrhea <500 mL/d or <3 episodes/d for adults
1	Maculopapular rash <25% of BSA	Bilirubin 2–3 mg/dL	Persistent nausea, vomiting, or anorexia	Diarrhea 500–999 mL/d or 3–4 episodes/d for adults
2	Maculopapular rash 25%–50% of BSA	Bilirubin 3.1–6 mg/dL	NA	Diarrhea 1000–1500 mL/d or 5–7 episodes/d for adults
3	Maculopapular rash >50% of BSA	Bilirubin 6.1–15 mg/dL	NA	Diarrhea >1500 mL/d or >7 episodes/d for adults
4	Generalized erythroderma (>50% BSA) plus bullous formation and desquamation >5% of BSA	Bilirubin >15 mg/dL	NA	Severe abdominal pain with or without ileus or grossly bloody stools (regardless of stool volume)
**Modified Glucksberg/Keystone Criteria[1,3]**				
Stage	Skin	Liver	Upper GI	Lower GI
0	No rash	Bilirubin <2 mg/dL	No persistent nausea and no histologic evidence of GVHD in the stomach or duodenum	Diarrhea <500 mL/d for adults
1	Rash <25% of BSA	Bilirubin 2–3 mg/dL	Persistent nausea with histologic evidence of GVHD in the stomach or duodenum	Diarrhea >500 mL/d for adults or persistent nausea
2	Rash 25%–50% of BSA	Bilirubin 3–6 mg/dL	NA	Diarrhea >1000 mL/d for adults
3	Rash >50% of BSA	Bilirubin 6–15 mg/dL	NA	Diarrhea >1500 mL/d for adults
4	Generalized erythroderma with bullous formation	Bilirubin >15 mg/dL	NA	Severe abdominal pain with or without ileus
**IBMTR Criteria[4]**				
Stage	Skin	Liver	Upper GI	Lower GI
0	No rash	Bilirubin <2 mg/dL	No persistent nausea and no histologic evidence of GVHD in the stomach or duodenum	Diarrhea <500 mL/d
1	Rash <25% of BSA	Bilirubin 2–3 mg/dL	Persistent nausea with histologic evidence of GVHD in the stomach or duodenum	Diarrhea >500 mL/d
2	Rash 25%–50% of BSA	Bilirubin 3.1–6 mg/dL	NA	Diarrhea >1000 mL/d
3	Rash >50% of BSA	Bilirubin 6.1–15 mg/dL	NA	Diarrhea >1500 mL/d
4	Bullae	Bilirubin >15 mg/dL	NA	Severe pain and ileus

aGVHD, acute GVHD; BSA, body surface area; GI, gastrointestinal; GVHD, graft-versus-host disease; IBMTR, International Bone Marrow Transplant Registry; MAGIC, Mount Sinai Acute GVHD International Consortium; NA, not applicable.

1. Schoemans HM, et al. EBMT-NIH-CIBMTR Task Force position statement on standardized terminology & guidance for graft-versus-host disease assessment. *Bone Marrow Transplant*. 2018;53(11):1401–1415.

2. Harris AC, et al. International, multicenter standardization of acute graft-versus-host disease clinical data collection: a report from the Mount Sinai Acute GVHD International Consortium. *Biol Blood Marrow Transplant*. 2016;22:4–10.

3. Przepiorka D, et al. 1994 Consensus conference on acute GVHD grading. *Bone Marrow Transplant*. 1995;15:825–828.

4. Rowlings PA, et al. IBMTR Severity Index for grading acute graft-versus-host disease: retrospective comparison with Glucksberg grade. *Br J Haematol*. 1997;97:855–864.

Figure reproduced from Sabatelli L, Keränen M, Viayna E, et al. Burden of hospitalizations and outpatient visits associated with moderate and severe acute graft-versus-host disease in Finland and Sweden: a real-world data analysis. *Support Care Cancer*. 2022. doi: 10.1007/s00520-022-06915-9, under the terms of the Creative Commons Attribution 4.0 International License (CC BY 4.0; https://creativecommons.org/licenses/by/4.0/), no modifications were made.

Supplementary Figure S2 Mapping Rules for Comparing MAGIC Grading to Other Scales

	Original Glucksberg			Modified Glucksberg			IBMTR		
MAGIC	Grade	Requirements for Inclusion in MAGIC II–IV	Exclusion	Grade	Requirements for Inclusion in MAGIC II–IV	Exclusion	Grade	Requirements for Inclusion in MAGIC II–IV	Exclusion
Grade II–IV	0	Upper GI=1	Upper GI=0	0	NEVER	ALWAYS	–	–	–
	I	Upper GI=1	Upper GI=0	I	NEVER	ALWAYS	A	NEVER	–
	II	–	–	II	–	–	B	Liver=1–2 and/or GI=1–2	B grade with only skin=2
	III	–	–	III	–	–	C	–	–
	IV	–	–	IV	–	–	D	–	–
Grade III/IV	II	GI=2 and/or liver=2	GI=0 and liver=1 OR GI=1 and liver=0 OR liver=1 and GI=1	II	NEVER	ALWAYS	B	Liver=2 and/or GI=2	Skin=2 (GI ≤1 and liver ≤1)
	III	–	–	III	–	–	C	Liver=2–3 and/or GI=2–3	Skin=3 (GI ≤1 and liver ≤1)
	IV	–	–	IV	–	–	D	–	–

GI, gastrointestinal; GVHD, graft-versus-host disease; IBMTR, International Bone Marrow Transplant Registry; MAGIC, Mount Sinai Acute GVHD International Consortium.

Figure reproduced from Sabatelli L, Keränen M, Viayna E, et al. Burden of hospitalizations and outpatient visits associated with moderate and severe acute graft-versus-host disease in Finland and Sweden: a real-world data analysis. *Support Care Cancer*. 2022. doi: 10.1007/s00520-022-06915-9, under the terms of the Creative Commons Attribution 4.0 International License (CC BY 4.0; https://creativecommons.org/licenses/by/4.0/), no modifications were made.

Supplementary Figure S3 Overview of aGVHD Grading Systems

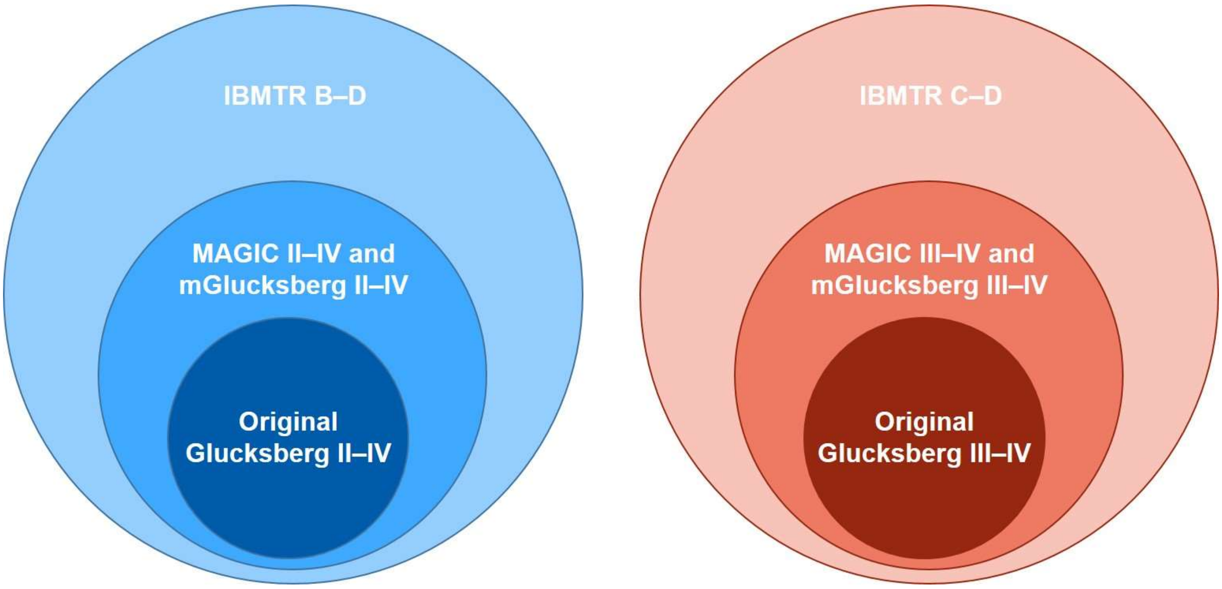

aGVHD, acute graft-versus-host disease; IBMTR, International Bone Marrow Transplant Registry; MAGIC, Mount Sinai Acute GVHD International Consortium; mGlucksberg, modified Glucksberg.

Figure reproduced from Sabatelli L, Keränen M, Viayna E, et al. Burden of hospitalizations and outpatient visits associated with moderate and severe acute graft-versus-host disease in Finland and Sweden: a real-world data analysis. *Support Care Cancer*. 2022. doi: 10.1007/s00520-022-06915-9, under the terms of the Creative Commons Attribution 4.0 International License (CC BY 4.0; https://creativecommons.org/licenses/by/4.0/), no modifications were made.

SUPPLEMENTARY TABLES

Supplementary Table S1. aGVHD Severity at Diagnosis Based on MAGIC and mGlucksberg Criteria

**Grading Scale**	**Finland (n=45)**	**Sweden (n=10)**	**France (n=96)**	**Overall (N=151)**
MAGIC grade, n (%)	n=25	n=4	n=0	n=29
I	0	0	0	0
II	20 (80.0)	1 (25.0)	0	21 (72.4)
III	5 (20.0)	3 (75.0)	0	8 (27.6)
IV	0	0	0	0
mGlucksberg grade, n (%)	n=20	n=6	n=96	n=122
I	0	0	12 (12.5)	12 (9.8)
II	13 (65.0)	5 (83.3)	46 (47.9)	64 (52.5)
III	7 (35.0)	0	17 (17.7)	24 (19.7)
IV	0	1 (16.7)	13 (13.5)	14 (11.5)
Unknown	0	0	8 (8.3)	8 (6.6)
Any scale, n (%)	n=45	n=10	n=96	N=151
I	0	0	12 (12.5)	12 (7.9)
II	33 (73.3)	6 (60.0)	46 (47.9)	85 (56.3)
III	12 (26.7)	3 (30.0)	17 (17.7)	32 (21.2)
IV	0	1 (10.0)	13 (13.5)	14 (9.3)
Unknown	0	0	8 (8.3)	8 (5.3)

aGVHD, acute graft-versus-host disease; MAGIC, Mount Sinai Acute GVHD International Consortium; mGlucksberg, modified Glucksberg.
